# Effect of Birth Preparedness on Institutional Delivery in Semiurban Ethiopia: A Cross-Sectional Study

**DOI:** 10.5334/aogh.920

**Published:** 2019-03-21

**Authors:** Carina Rosado, Jennifer A. Callaghan-Koru, Abiy Estifanos, Ephrem Sheferaw, Thewodros Shay, Joseph de Graft-Johnson, Barbara Rawlins, Hannah Gibson, Abdullah H. Baqui, Bareng Aletta Sanny Nonyane

**Affiliations:** 1 Deloitte LLP, US; 2 University of Maryland, Baltimore County, US; 3 Addis Ababa University, ET; 4 Maternal and Child Health Integrated Program, ET; 5 Maternal and Child Health Integrated Program, US; 6 Johns Hopkins University Bloomberg School of Public Health, US

## Abstract

**Background:**

Ethiopia has one of the lowest rates of facility delivery and is promoting birth preparedness among pregnant women through its community health services to increase the rate of institutional delivery and reduce maternal mortality. Observational studies of birth preparedness in Ethiopia have thus far only reported the marginal effect of birth preparedness when controlling for other factors, such as parity and education.

**Objectives:**

In this cross-sectional study, we use propensity score modeling to estimate the average population-level effect of birth preparedness on the likelihood of delivering at a facility.

**Methods:**

We conducted secondary analysis of household survey data collected from 215 women with a recent live birth within the catchment areas of 10 semi-urban health centers. A mother was considered well prepared for birth if she reported completing four of the following six actions: identified a skilled provider, identified an institution, saved money, identified transport, prepared clean delivery materials, and prepared food. We performed unadjusted and multivariate logistic regression analyses, with and without propensity score weighting, to assess the relationship between birth preparedness and institutional delivery.

**Findings:**

One hundred respondents (47%) delivered in an institution, and over two-thirds (151, 71%) were considered well prepared for birth. Institutional delivery was more common among women who were considered well prepared (57%) versus those who were considered not well prepared (19%). In the model with propensity score weighting, women who were well prepared for birth had 3.83 times higher odds of delivering at a facility (95% CI: 1.41–10.40, p-value = 0.010).

**Conclusions:**

This study contributes to existing evidence supporting the inclusion of antenatal birth preparedness counseling as a part of an antenatal care package for promoting institutional delivery. Important gaps remain in operationalizing the definition of birth preparedness and understanding the pathway from exposure to outcome.

## Introduction

Globally, over 300,000 mothers die annually due to pregnancy- and childbirth-related complications. Ninety-nine percent of these deaths occur in developing countries, with Sub-Saharan Africa absorbing over half (66%) of this burden [1]. The majority of maternal deaths occur during labor, delivery, and in the first 24 hours postpartum; most complications cannot be predicted or prevented [2]. Timely diagnosis and appropriate management of complications require considerable skill of those providing care; thus, where a woman gives birth, the delivery attendant’s skill and the proximity to a referral care center are critical factors influencing maternal survival [3]. Giving birth in a health institution has been suggested as the single most effective intervention to end preventable maternal deaths [3]; however, there is limited evidence on interventions that increase institutional delivery rates. As the global community seeks to operationalize the Sustainable Development Goal 3 targets to reduce maternal mortality and end preventable newborn deaths, it is imperative to critically assess what strategies accelerate the rate and quality of institutional deliveries.

One of the strategies to increase the coverage of skilled attendance at birth is the promotion of birth preparedness among pregnant women. The “three delays” model describes three junctures at which delayed care can result in maternal deaths: delay in deciding to seek medical care, delay in reaching medical care, and delay in receiving care [4]. Birth preparedness is considered a promising strategy to decrease one or all of the delays, thereby increasing the likelihood of surviving obstetric emergencies [5]. Birth preparedness has been conceptualized to span knowledge and awareness, intentions, and actions taken by pregnant women and their families, providers, and community, which affect the timely and appropriate access to obstetric care [6]. The concept of birth preparedness is operationalized with varying definitions in practice and in the literature. In the World Health Organization’s Integrated Management of Pregnancy and Childbirth guidelines, the standard for birth and emergency readiness is that “all women should have a written plan for birth and for dealing with unexpected adverse events,” and health providers are expected to educate women about signs of labor and danger and support women and communities in developing their plans [7]. The implicit assumption of this model is that birth preparedness counseling provided by health workers will lead to birth preparedness actions taken by stakeholders. That is, by knowing the risks and danger signs, having the intention to undertake preparatory actions, and completing preparatory actions, pregnant women will be more likely to deliver in a health institution and have access to emergency obstetric care in the event of a complication. Despite the promise of birth preparedness for increasing facility delivery, few trials have tested the effectiveness of birth preparedness [8] —more often, birth preparedness counseling is included in a package of interventions, making it difficult to isolate the effect of birth preparedness [9,10]. The evidence for effectiveness of birth preparedness is also limited by geography—given that factors influencing both birth preparedness and facility delivery are present at individual, community, and health system and policy levels [11], there are limitations in generalizing results from one country to others.

Ethiopia, an East African country with one of the lowest global coverage rates of skilled attendance at delivery (27%) [12], has incorporated birth preparedness counseling into its community health services in an effort to increase facility delivery [13]. There is a growing literature of observational studies in Ethiopia assessing the status of birth preparedness in various regions [14,15,16,17,18,19,20,21,22,23,24] and associations between birth preparedness and facility delivery [25,26,27,28,29] or skilled birth attendance [30]. Unlike the results generated by an experimental design, which report the average population-level effect of an intervention, these predominantly retrospective observational studies rely on multivariate regression analyses, which estimate the *marginal* effect of birth preparedness beyond other factors influencing facility delivery that are included in the statistical model [31]. Given that no trial of birth preparedness has taken place in Ethiopia, the population level increase in facility delivery that can be expected from improved birth preparedness is unknown. This study addresses that gap by using propensity score analysis to estimate the average population level effect of birth preparedness on facility delivery, when controlling for measured confounders among a sample of new mothers in four regions of Ethiopia.

## Methods

### Study Design

For this study we conducted secondary analysis of household survey data collected for an evaluation of community-based promotion of skin-to-skin care (SSC) and exclusive breastfeeding by Health Extension Workers (HEWs) in Ethiopia, the results of which are published elsewhere [47]. While promoting birth preparedness is part of an HEW’s stated responsibilities [13], this study did not target improving birth preparedness and did not include training on birth preparedness counseling as part of the study protocol.

### Data source

The household survey, for which detailed methods have been published [47,48], took place between December 2013 and January 2014 in four regions—Oromia, Tigray, Amhara, and Southern Nations, Nationalities, and People (SNNP)—which account for 85% of the country’s population and represent the cultural and ethnic diversity in Ethiopia. Women who reported a live birth within the 7 months prior to data collection were eligible for the survey. Respondents were sampled from 34 randomly-selected census enumeration areas surrounding the ten health centers following a probability proportional to size approach. A total of 7,669 households were screened, 337 eligible women were identified, and 215 were randomly selected to complete the survey according to the sample size requirements for the evaluation study. Data were collected using a knowledge, practice, and coverage questionnaire developed by the Saving Newborn Lives program and adapted for this evaluation.

### Variables

The primary outcome variable for this analysis is institutional delivery, which includes delivery in a government hospital, government health center, nongovernmental organizational clinic, or private hospital. The main predictor variable is a composite indicator for completing birth preparedness actions. Researchers and practitioners have varying interpretations of the knowledge, intentions, and behavioral elements that constitute birth preparedness and with which combination of these a woman is considered well prepared for birth. We narrowly defined birth preparedness in terms of specific actions or behavioral practices the mothers reported undertaking during pregnancy. Following a similar approach as that in other studies [15,16,18,22,23,26,32], we created a composite indicator of individual birth preparedness actions and assigned a cutoff value to classify a respondent as being well prepared for birth versus not well prepared for birth.

Our definition of birth preparedness was developed through a review of 17 peer-reviewed papers on birth preparedness in Ethiopia and similar settings (see Supplemental Table 1), a review of the HEW program guidelines on birth preparedness counseling [33], and consideration of the variables measured in the survey. We classified recently delivered women as well prepared for birth if they reported completing at least four of the following six actions related to delivery: (1) identified a skilled provider, (2) identified an institution for delivery, (3) saved money for delivery, (4) identified transport, (5) prepared delivery materials, and (6) made provisions for food (see Panel 1). The first four items in this index are also included in the majority of published birth preparedness indices and scales that we reviewed. The fifth item, preparation of delivery materials, is not included in all indices, but is included in the HEW training guidelines for birth preparedness counseling [13]. The sixth item, food preparation, is only included in two prior indices we reviewed [28,34]. We decided to include this item in our definition of birth preparedness because it was an action measured in our survey and was completed by the majority of women; in sensitivity analyses, the removal of food preparation from the index only changed the preparedness classification of 3 (1%) of the women in the sample. We selected a threshold of 4 items for classification as “well prepared” because the mean and median number of the six actions that women in our sample completed was 4, and the majority of reviewed studies classified women as prepared if they complete more than half of measured actions (Supplemental Table 1).

**Panel 1:** Birth preparedness practices.


The individual birth preparedness practices included are:
***Identified skilled provider:*** During her pregnancy, woman planned to have a skilled health worker attend the birth of her child.
***Identified facility for delivery:*** During her pregnancy, woman identified a health facility to deliver her child.
***Saved money:*** Woman or family set aside funds specifically for care during delivery.
***Identified transport:*** During her pregnancy, woman prepared a means of transportation for delivery.
***Identified delivery materials:*** During her pregnancy, woman prepared materials for clean delivery. Materials may include soap and water for washing hands, new blade to cut the umbilical cord and a sterilized thread to tie the cord, clean cloth to wipe and wrap the baby, clean space, and a carpet or mat for the delivery.
***Preparation for food:*** During her pregnancy, woman made food provisions for her delivery.


Sociodemographic and health care utilization variables that we considered as potential confounders were maternal age, marital status, parity, educational level, household wealth level, knowledge of pregnancy danger signs, distance to a health center, and time of labor start. We used principal component analysis to produce a wealth score for each household using housing material, toilet source, drinking water source, and household assets. A woman was considered knowledgeable about pregnancy risks if she could name at least one of the five danger signs without being prompted [35]: fever, bleeding, convulsions, swelling of hands and feet, or ruptured membranes before term.

### Statistical analysis

We included all 215 respondents in the dataset to analyze the effect of birth preparedness on institutional delivery. Descriptive statistics for the prevalence of birth preparedness actions were calculated adjusting for survey design. In order to estimate a population-level effect of birth preparedness on facility delivery—rather than the marginal effect estimated by multivariate regression models—we used propensity scoring methods to improve the comparability of the treatment groups on characteristics we could measure. This propensity score analysis uses the weighting approach, which provided better balance between treatment groups than a full matching approach [36] and was conducted in two stages. In the first stage, we fitted a propensity score model with treatment (birth preparedness) as the outcome and background covariates as predictors (age, marital status, schooling level, parity, household wealth, knowledge of pregnancy danger signs, time of labor start, distance to health center, and sample weight). We used a logistic regression model and took as propensity scores the estimates of the probability of a woman receiving the treatment (birth preparedness = yes). In the second stage, we fitted and weighted the outcome model (delivering in an institution) by the propensity score [36]. In the unadjusted and adjusted models, we present results with and without the sample weights. Per DuGoff et al., we generated a composite score that comprised the propensity score multiplied by the sample weights to estimate an average treatment effect generalizable to the target population [37]. Furthermore, these models were extended to include the covariates (e.g., age, marital status, etc.) to account for any residual confounding [37]. We assessed the multicollinearity between the independent variables using a variance inflation factor for survey data. All analyses were conducted using Stata 13.1 [38].

## Results

*Sociodemographic characteristics.* Table 1 provides a summary of the background characteristics of the 215 respondents. Overall, 77 (36%) respondents were from Amhara, 60 (28%) from Oromia, 64 (30%) from SNNP, and 14 (7%) from Tigray regions. Half of the respondents (111, 52%) were 25–34 years of age, and almost all women (201, 94%) were married or living with a partner. Eighty-seven (41%) respondents had no schooling, 88 (41%) had completed primary school, and the remaining 38 (18%) respondents had completed at least secondary school. The respondents were about evenly distributed among the Ethiopian Christian Orthodox (81, 38%), Protestant (65, 30%), and Muslim (68, 32%) religious affiliations. The most common ethnicities were Amhara (88, 41%), Oromo (38, 18%), and Hadiya (34, 16%). Results of the unweighted and unadjusted logistic regression, which are in Table 1, indicate that there are statistically significant differences in maternal education and wealth level among women who delivered in an institution compared with those who did not.

**Table 1:** Characteristics of the sample and their association with facility delivery.

	Total	Nonfacility delivery	Facility delivery	Unadjusted analysis^a^		

	n (%) n = 215	n (%) n = 115	n (%) n = 100	OR^b^	P-value	95% CI^b^

Region						

	n = 215	n = 115	n = 100			
SNNP	64 (30%)	48 (42%)	16 (16%)	1.00		
Oromia	60 (28%)	29 (25%)	31 (31%)	3.21	0.064	0.93–11.03
Tigray	14 (7%)	8 (7%)	6 (6%)	2.25	0.521	0.18–28.64
Amhara	77 (36%)	30 (26%)	47 (47%)	4.70	0.028	1.19–18.49
Age of Child						

	n = 214	n = 114	n = 100			
2–10 weeks	52 (24%)	25 (22%)	27 (27%)	1.00		
11–20 weeks	101 (47%)	49 (43%)	52 (52%)	0.98	0.955	0.53–1.83
21–30 weeks	61 (29%)	40 (35%)	21 (21%)	0.49	0.065	0.23–1.04
Sex of child						

	n = 214	n = 114	n = 100			
Female	107 (50%)	63 (55%)	44 (44%)	1.00		
Male	108 (50%)	52 (45%)	56 (56%)	1.54	0.177	0.81–2.92
Age of respondent (mother)						

	n = 213	n = 113	n = 100			
15–24 years	69 (33%)	32 (28%)	37 (37%)	1.00		
25–34 years	111 (52%)	61 (54%)	40 (40%)	0.71	0.353	0.34–1.49
35 years or older	33 (15%)	20 (18%)	13 (13%)	0.56	0.188	0.24–1.34
Marital status						

	n = 213	n = 113	n = 100			
Currently married/living together	201 (94%)	110 (97%)	91 (91%)	1.00		
Formerly married or never married	12 (6%)	3 (3%)	9 (9%)	3.63	0.064	0.92–14.23
Maternal education						

	n = 213	n = 115	n = 98			
None	87 (41%)	62 (54%)	25 (26%)	1.00		
Primary	88 (41%)	46 (40%)	42 (43%)	2.26	0.036	1.06–4.85
Secondary or higher	38 (18%)	7 (6%)	31 (32%)	10.98	0.000	3.61–33.42
Religion						

	n = 215	n = 115	n = 100			
Orthodox	81 (38%)	40 (35%)	41 (41%)	1.00		
Protestant	65 (30%)	43 (38%)	22 (22%)	0.50	0.243	0.15–1.64
Muslim	68 (32%)	32 (28%)	36 (36%)	1.10	0.848	0.41–2.92
Other	1 (0%)	0 (0%)	1 (1%)	N/A^b^	N/A	N/A
Ethnicity						

	n = 215	n = 115	n = 100			
Hadiya	34 (16%)	26 (23%)	8 (8%)	1.00		
Oromo	38 (18%)	22 (19%)	16 (16%)	2.36	0.230	0.56–9.89
Amhara	88 (41%)	35 (30%)	53 (53%)	4.92	0.037	1.10–21.96
Gamo	23 (11%)	13 (11%)	10 (10%)	2.50	0.367	0.33–19.19
Other	32 (15%)	19 (17%)	13 (10%)	2.22	0.352	0.40–12.45
Wealth score						

	n = 213	n = 115	n = 108			
Quintile 1	44 (21%)	41 (36%)	3 (3%)	1.00		
Quintile 2	42 (20%)	32 (28%)	10 (9%)	4.27	0.040	1.08–16.94
Quintile 3	42 (20%)	21 (19%)	33 (30%)	13.67	0.001	3.44–54.30
Quintile 4	44 (21%)	11 (10%)	31 (29%)	41.0	<0.001	9.83–171.0
Quintile 5	41 (20%)	10 (9%)	31 (29%)	42.36	<0.001	10.11–177.6
Parity						

	n = 210	n = 112	n = 98			
First birth	65 (31%)	23 (21%)	42 (43%)	1.00		
2–4 births	104 (50%)	58 (52%)	46 (47%)	0.43	0.005	0.25–0.76
5 or more births	41 (20%)	31 (28%)	10 (10%)	0.18	<0.001	0.08–0.42
Number of ANC visits						

	n = 214	n = 114	n = 100			
Less than 4	105 (49%)	74 (65%)	31 (31%)	1.00		
4 or more	109 (51%)	40 (35%)	69 (69%)	4.12	<0.001	2.28–7.43
Time of labor start						

	n = 204	n = 108	n = 96			
Day (between 6 am to 5 pm)	99 (49%)	47 (44%)	52 (54%)	1.00		
Night (between 6 pm to 5 am)	105 (52%)	61 (56%)	44 (46%)	0.65	0.219	0.33–1.31
Distance to health center						

	n = 214	n = 114	n = 100			
Less than 30 minutes	42 (20%)	8 (7%)	34 (34%)	1.00		
30–59 minutes	64 (30%)	23 (20%)	41 (41%)	0.42	0.130	0.13–1.31
More than 1 hour	108 (51%)	83 (73%)	25 (25%)	0.07	<0.001	0.02–0.21
Knowledge of pregnancy danger signs						

	n = 210	n = 110	n = 100			
Know at least one danger sign	147 (70%)	69 (63%)	78 (78%)	1.00		
Do not know any danger signs	63 (30%)	41 (37%)	22 (22%)	2.11	0.055	0.98–4.52

^a^ Unadjusted analysis using logistic regression accounting for clustering by enumeration area. ^b^ Confidence interval (CI), odds ratio (OR), not available (N/A).

*Use of reproductive health services and other obstetric characteristics.* One hundred (47%) respondents gave birth in an institution. About one-third of the respondents (65, 31%) were primipara mothers, half had given birth to 2–4 children (104, 50%), and the remaining 41 respondents (20%) had five or more children. The respondents were about evenly divided between those who had or had not received four or more ANC visits by the time of labor start. Half of the respondents lived more than one hour from the nearest health center (106, 51%). The great majority (147, 70%) of the respondents knew at least one danger sign. The results of the unweighted and unadjusted logistic regression (Table 1) show statistically significant associations between parity, four or more ANC visits, and distance to health center and the outcome of institutional delivery.

*Status of birth preparedness.* Table 2 provides the unweighted prevalence of each birth preparedness practice among the 215 respondents. Most (195, 91%) reported making provisions for food during pregnancy, 171 (80%) reported making provisions for clean delivery materials, 153 (71%) saved money, 152 (71%) planned to deliver in an institution, 143 (67%) reported identifying a skilled birth attendant, and 84 (39%) reported making preparations for transport. The median amount of money saved among respondents who reported saving money was ETB 1,000 (about USD 52). When sample weights were applied, the weighted and unweighted prevalence for four of the six birth preparedness practices remained similar (within 2% of the unweighted percentages). The two practices with a lower weighted prevalence were respondents saving money (70%) and respondents making preparations for transport (32%). The unadjusted analysis showed significant differences between women who delivered outside of an institution and women who delivered at an institution, with respect to four practices: planning for an institutional delivery, identifying a birth attendant, saving money, and preparing transport.

**Table 2:** Coverage of birth preparedness actions and their association with facility delivery.

	Total	Nonfacility delivery	Facility delivery	Unadjusted analysis^a^		

N = 215	n (%)	n (%)	n (%)	OR^b^	P-value	95% CI^b^

Planned to deliver in an institution^c^						

	n = 213	n = 115	n = 98			
No	61 (29%)	50 (43%)	11 (11%)	1.00		
Yes	152 (71%)	65 (57%)	87 (89%)	6.08	0.000	2.44–15.15
Planned for skilled birth attendant						

	n = 215	n = 115	n = 100			
No	72 (33%)	55 (48%)	17 (17%)	1.00		
Yes	143 (67%)	60 (52%)	83 (83%)	4.48	0.001	1.96–10.22
Saved money						

	n = 215	n = 115	n = 100			
No	62 (29%)	41 (36%)	21 (21%)	1.00		
Yes	153 (71%)	74 (64%)	79 (79%)	2.08	0.024	1.11–3.92
Prepared transport						

	n = 215	n = 115	n = 100			
No	131 (61%)	81 (70%)	50 (50%)	1.00		
Yes	84 (39%)	34 (30%)	50 (50%)	2.38	0.019	1.17–4.86
Prepared clean delivery materials						

	n = 215	n = 115	n = 100			
No	44 (21%)	28 (24%)	16 (16%)	1.00		
Yes	171 (80%)	87 (76%)	84 (84%)	1.68	0.179	0.78–3.67
Prepared food						

	n = 215	n = 115	n = 100			
No	20 (9%)	12 (10%)	8 (8%)	1.00		
Yes	195 (91%)	103 (90%)	92 (92%)	1.34	0.595	0.44–4.05
Well prepared^d^						

	n = 213	n = 115	n = 98			
No	62 (29%)	50 (44%)	12 (12%)	1.00		
Yes	151 (71%)	65 (57%)	86 (88%)	5.51	<0.001	2.41–12.57

^a^ Unadjusted analysis using logistic regression accounting for clustering by enumeration area. ^b^ Confidence interval (CI), odds ratio (OR). ^c^ Missing two values. ^d^ A mother was considered well prepared for birth if she reported completing at least four of the following six actions in advance of her delivery: identified a skilled provider, identified an institution, saved money, identified transport, prepared clean delivery materials, and prepared food.

Table 2 also includes the unweighted prevalence of respondents who were considered well prepared, as defined by taking action on at least four birth preparedness factors. More than two-thirds of the respondents (151, 71%) were categorized as well prepared for birth. Among respondents who were well prepared for birth, more respondents delivered in an institution (n = 86, 57%); among the 62 respondents who were not well prepared for birth, only 12 delivered in an institution (1%). When we applied sampling weights, the percentage of respondents delivering in an institution, from among those who were well prepared, decreased from 57% (unweighted) to 50% (weighted); the percentage of respondents delivering in an institution from among women who were not well prepared remained at 19%.

*Association between birth preparedness and institutional delivery.* Table 3 presents four analyses assessing the association between birth preparedness and institutional delivery.

**Table 3:** Logistic regression estimates of the effect of birth preparedness on the odds of institutional delivery.

Unadjusted analysis				Multivariate analysis		

N = 186^a^	OR^b^	95% CI^c^	P-value	OR	95% CI	P-value

Well prepared (without propensity score weighting^d^)						
		**Model 1**			**Model 3**	
**Yes vs. no**	5.47	2.16–13.88	0.001	3.20	1.20–8.51	0.022
Well prepared (with propensity score weighting^d^)						
		**Model 2**			**Model 4**	
**Yes vs. no**	3.83	1.41–10.40	0.010	4.56	1.71–12.14	0.003

^a^ All models are limited to complete cases only to facilitate comparison across models. ^b^ Odds ratio (OR). ^c^ Confidence interval (CI). ^d^ Propensity score includes mother’s age, marital status, parity, educational level, wealth status, ANC visits, knowledge of pregnancy danger signs, distance to health center, time of labor start, and sample weight.

Model 1. This model provides an unadjusted analysis without propensity score weighting. The results suggest that women who completed four or more birth preparedness steps, and were thus considered well prepared for birth, were at least five times more likely to have delivered in an institution compared with women who were not well prepared (OR = 5.47, 95% CI: 2.16–13.88, p-value = 0.001). The results of Model 1 with sample weights applied were OR = 4.22, 95% CI: 1.61–11.05, p-value = 0.005.

Model 2. An extension of Model 1, we controlled for potential confounding using propensity score weighting for the unadjusted outcome. The results suggest that women who were well prepared were at least three times more likely to have given birth in an institution (OR = 3.83, 95% CI: 1.41–10.40, p-value = 0.010) compared with women who were not well prepared. When we applied a composite weight (propensity score multiplied by sample weight), the results were OR = 3.44, 95% CI: 1.18–10.02, p-value = 0.025.

Model 3. This model assessed the association between institutional delivery and birth preparedness and adjusted for other potential confounding covariates using multivariate logistic regression without propensity score weighting. Women who were well prepared were at least three times more likely (OR = 3.20, 95% CI: 1.20–8.51, p-value = 0.022) to deliver in an institution compared with women who were not well prepared. The results of Model 3 with sample weights applied were OR = 3.06, 95% CI: 1.11–8.39, p-value = 0.031.

Model 4. An extension of Model 3, Model 4 looked at the association between institutional delivery and birth preparedness using a multivariate logistic regression model with propensity score weighting. The results suggest that well-prepared women were at least four times more likely to have delivered in an institution (OR = 4.56, 95% CI: 1.71–12.14, p-value = 0.003) compared with women who were not well prepared. When we applied a composite weight of propensity score multiplied by sample weight, the results were OR = 4.84, 95% CI: 1.62–14.51, p-value = 0.006.

## Discussion

This study provides what we believe is the first estimate of the average population-level effect of birth preparedness on facility delivery in Ethiopia. Across models, well-prepared women were at least three times more likely to give birth in a facility compared with women who were not well prepared. In fact, the estimated average population-level treatment effect of birth preparedness reported in the propensity score models (2 and 4) was similar in direction and magnitude to the marginal effect of birth preparedness reported in the standard multivariate regression models (1 and 3). In bivariate analyses, planning to deliver in a health facility and with a skilled attendant were the two birth preparedness components with the strongest association with facility delivery among the six that we measured.

This study also contributes to the rapidly growing literature on the prevalence of birth preparedness actions among pregnant women in Ethiopia. A large proportion of respondents (71%), who were drawn from a semi-urban population with health indicators generally higher than women in a primarily rural sample [12], were well prepared for birth. Two earlier studies—one covering the same regions [17] and another in the Tigray region [19] —reported very similar results: close to 70% of pregnant women were classified as well prepared for birth. Several other studies, from regions including Oromia, SNNP, Tigray, and Amharra, reported much lower rates of birth preparedness between 13% and 38% [15,16,20,21,22,23,24,28,30,39].

While we found a positive association between birth preparedness and institutional delivery, previous research on the effectiveness of birth preparedness promotion and care-seeking has been mixed. Studies in Ethiopia, Tanzania, and Nepal reported positive associations between birth preparedness and institutional delivery. Two unmatched case-control studies in Ethiopia reported positive marginal effects of birth preparedness on facility delivery: one study reported a six times greater odds for institutional delivery (adjusted OR (AOR) = 6.96, 95% CI: 2.42–19.99, p-value not provided) [25]; and another reported a three times greater odds for institutional delivery (AOR = 2.55, 95% CI: 1.12–5.84, p-value < 0.05) [27]. In Tanzania, a randomized control trial reported a 17 percentage point increase in facility delivery among women who were counseled on birth plans compared with women who did not receive counseling (adjusted difference in proportions: 16.8%, 95% CI: 2.6–31.0, p-value = 0.02) [40], and a cross-sectional study reported that well-prepared women were almost four times more likely to deliver in an institution (AOR = 3.91, 95% CI: 2.44–6.27, p-value not provided) [32]. A prospective, observational study in Nepal reported a slightly positive association between more birth preparedness arrangements taken and skilled attendance at birth (OR = 1.52, 95% CI: 1.22–1.88, p < 0.001) [41]. Other studies exploring the association between birth preparedness and skilled attendant or institutional delivery have found weak or no association between the two constructs. One prospective, observational study in Ethiopia reported a weak positive marginal association between birth preparedness and skilled attendance at birth (AOR = 1.32, 95% CI: 1.03–1.68, p-value not provided) [26], and a cross-sectional study reported no association (AOR 1.9, 95% CI: 0.9–4.1, p 0.091) [29]. In India, Burkina Faso, and Nepal, studies found women prepared for birth were no more likely to give birth with the assistance of a skilled provider or in an institution than women who were not prepared [8,42,43].

Among the studies from Ethiopia, there are a number of potential reasons for the variations in the rate of birth preparedness and its association with institutional delivery. First, differences in the make-up of the samples, with regard to variables reported to be predictors of birth preparedness, could be an influence. Residence, educational status, ANC attendance, parity, wealth level, knowledge of pregnancy danger signs, and past obstetric complications are some variables associated with an increased likelihood for preparing for birth [14,15,1,18,19,20,21,23]. Second, the studies have been conducted over the course of the last decade and in different regions in Ethiopia. The relatively recent emphasis on birth preparedness and the role of HEWs in communicating birth preparedness messages could be contributing to the difference in rates. While there is national-level guidance on promotion of birth preparedness by health care workers at the community and facility levels, it is not well understood to what extent women are receiving these messages and from whom. Differences in source, frequency, and consistency of the messaging could explain differences in prevalence of birth preparedness.

In Ethiopia and elsewhere, an important consideration for interpreting the results of birth preparedness studies is the varying definitions of birth preparedness used by each study, both in terms of what elements are included and how many actions a woman must complete to be considered well prepared. While the concept of birth preparedness encompasses knowledge, intentions, and behaviors, researchers have differed in their operational definitions and measurements of birth preparedness by focusing on select elements within each, some, or all of the components. As was done in this present study, some researchers treat knowledge as a preceding step to birth preparedness actions; thus, they do not include knowledge within the primary independent variable (birth preparedness) [14,15,18,21,44]. Some researchers have chosen to include the number of ANC visits, the content of the visits, or both, as part of the birth preparedness variable [8,41]. Another issue is the distinction that is inconsistently applied between birth preparedness and complication readiness [45]. While the two concepts are related, birth preparedness is about encouraging women and their families to make preparations for all deliveries, while complication readiness tends to focus more on raising awareness about signs of and responses to obstetric emergencies.

The definitional inconsistencies of birth preparedness prevent a clear understanding of the effectiveness of birth preparedness counseling and what specific actions lead to institutional delivery across studies and settings. Practitioners looking to translate research into public health programs would be rightfully confused when trying to determine which elements of birth preparedness have been shown to be effective and incorporate those elements into programs. Brazier et al. disassembled the concepts of birth preparedness knowledge, complication readiness knowledge, and birth preparedness actions, to create separate composite variables for the components of these two constructs [45]. In their sample of mothers from Guinea, birth preparedness knowledge and actions were associated with institutional delivery, while complication readiness knowledge was not. Future research should also consider whether the effective birth preparedness components differ between settings, requiring context-specific definitions of birth preparedness. For example, identifying transport may be more important in remote or sparsely populated rural areas than semi-urban or urban areas. Additionally, varying policies and informal practices within health systems also may determine the extent to which saving money or preparing clean delivery materials influence institutional delivery.

This study compared results, with and without sample weights, for propensity score weighting (Model 2) and regression-adjustment (Model 3), and we combined propensity score weighting with the regression-adjustment approach (Model 4) to control for error when estimating the association between birth preparedness and institutional delivery. Although all models in our analysis consistently found a positive association between birth preparedness and facility delivery, we observed extreme survey sample weights in the control group (birth preparedness = no). When propensity score weighting was combined with the composite weight (propensity score multiplied by the sample weight), this imbalance was further exaggerated, which resulted in additional variability in the measure of association, as seen in the results of Model 4. This is one of the known issues to propensity score weighting [36]. While propensity scores and regression adjustments are alternative approaches to controlling for background variables, these methods are complementary and indeed have been shown to work best when combined [36]. In this study, propensity score weighting with regression adjustment resulted in a stronger association yet added variability compared to either approach alone. Future work involving simulation studies will be useful to help understand the implications of using composite weights with small data sets.

Our study has several limitations. First, the study design is cross-sectional and relies on a woman’s retrospective, self-reported preparations for childbirth. Self-reports may be subject to increased recall bias as time elapses. However, the study participants were questioned within 7 months of giving of birth, which is a shorter time period than what has been reported (e.g., 1 year or 2 years) in many observational birth preparedness studies. Second, women’s retrospective, self-reported preparations for delivery may be influenced by the care they receive during childbirth [5]. Third, we did not assess actual or perceived quality of care provided by local health institutions for normal and emergency deliveries. Because clients’ perceptions of quality shape care-seeking behavior, differences in the quality of care delivered among health institutions could influence the results [46]. Fourth, identification of a blood donor is a birth preparedness practice commonly included in birth preparedness definitions, but the questionnaire we used did not measure this.

## Conclusion

The results from this study suggest that increasing birth preparedness behaviors during pregnancy will likely increase population-level coverage of facility delivery. These findings are supportive of the Ethiopian Ministry of Health’s efforts to promote birth preparedness as a key component of ANC provided by health care workers and the work to expand coverage of ANC so all women can receive and act on birth preparedness messaging. In this study, the elements included as a part of the composite variable for birth preparedness associated with facility delivery are identifying a skilled provider, identifying a health institution for birth and making arrangements to save money, identifying transport, identifying delivery materials, and preparing food.

## Additional File

The additional file for this article can be found as follows:

10.5334/aogh.920.s1 Supplemental Table 1. Definitions of Birth Preparedness in Previous Studies.

## Figures and Tables

**Table:** 

ANC	antenatal care
AOR	adjusted odds ratio
CI	confidence interval
HEW	health extension worker
OR	odds ratio
SSC	skin-to-skin care
SNNP	Southern Nations, Nationalities, and People

## References

[1] Alkema L, Chou D, Hogan D, Zhang S, Moller A-B and Gemmill A. Global, regional, and national levels and trends in maternal mortality between 1990 and 2015, with scenario-based projections to 2030: A systematic analysis by the UN Maternal Mortality Estimation Inter-Agency Group. Lancet. 2016; 387(10017): 462–474. DOI: 10.1016/S0140-6736(15)00838-7 26584737 PMC5515236

[2] Ronsmans C and Graham WJ. Maternal mortality: Who, when, where, and why. Lancet. 2006; 368(9542): 1189–1200. DOI: 10.1016/S0140-6736(06)69380-X 17011946

[3] Campbell OM and Graham WJ. Strategies for reducing maternal mortality: Getting on with what works. Lancet. 2006; 368(9543): 1284–1299. DOI: 10.1016/S0140-6736(06)69381-1 17027735

[4] Thaddeus S and Maine D. Too far to walk: Maternal mortality in context. Soc Sci Med. 1994; 38(8): 1091–1110. DOI: 10.1016/0277-9536(94)90226-7 8042057

[5] Stanton CK. Methodological issues in the measurement of birth preparedness in support of safe motherhood. Eval Rev. 2004; 28(3): 179–200. DOI: 10.1177/0193841X03262577 15130180

[6] Maternal and Neonatal Health (MNH) Program. Birth Preparedness and Complication Readiness: A Matrix of Shared Responsbilities. 2004: 1–12.

[7] World Health Organization. Integrated Management of Pregnancy and Childbirth. WHO Recommended Interventions for Improving Maternal and Newborn Health. 2009: 1–6. WHO/MPS/>07.05.

[8] McPherson RA, Khadka N, Moore JM and Sharma M. Are birth-preparedness programmes effective? Results from a field trial in Siraha District, Nepal. J Heal Popul Nutr. 2006; 24(4): 479–488. DOI: 10.1097/INF.0b013e31819069e8 PMC3001152 17591345

[9] Soubeiga D, Gauvin L, Hatem MA and Johri M. Birth Preparedness and Complication Readiness (BPCR) interventions to reduce maternal and neonatal mortality in developing countries: Systematic review and meta-analysis. BMC Pregnancy Childbirth. 2014; 14: 129 DOI: 10.1186/1471-2393-14-129 24708719 PMC4234142

[10] Miltenburg AS, Roggeveen Y, Shields L, et al. Impact of birth preparedness and complication readiness interventions on birth with a skilled attendant: A systematic review. PLoS One. 2015; 10(11): 1–21. DOI: 10.1371/journal.pone.0143382 PMC4658103 26599677

[11] Moyer CA and Mustafa A. Drivers and deterrents of facility delivery in sub-Saharan Africa: A systematic review. Reprod Health. 2013; 10(1). DOI: 10.1186/1742-4755-10-40 PMC3751820 23962135

[12] Central Statistical Agency (CSA) [Ethiopia] and ICF. Ethiopia Demographic and Health Survey 2016 Addis Ababa, Ethiopia, and Rockville, Maryland, USA; 2016 https://dhsprogram.com/pubs/pdf/FR328/FR328.pdf.

[13] Ministry of Health [Ethiopia]. Integrated Refresher Training for Health Extension Workers – Module II (CMNCH) Facilitator Guide Addis Ababaa, Ethiopia; 2011.

[14] Hiluf M and Fantahun M. Birth Preparedness and Complication Readiness among women in Adigrat town, north Ethiopia. Ethiop J Heal Dev. 2008; 22(1): 14–20. DOI: 10.4314/ejhd.v22i1.10057

[15] Hailu M, Gebremariam A, Alemseged F and Deribe K. Birth preparedness and complication readiness among pregnant women in Southern Ethiopia. PLoS One. 2011; 6(6). DOI: 10.1371/journal.pone.0021432 PMC3120869 21731747

[16] Zepre K and Kaba M. Birth preparedness and complication readiness among rural women of reproductive age in Aabeshige district, Gguraghe zone, SNNPR, Ethiopia. Int J Womens Health. 2017; 9: 11–21. DOI: 10.2147/IJWH.S111769 28053557 PMC5191624

[17] Karim AM, Admassu K, Schellenberg J, et al. Effect of Ethiopia’s Health Extension Program on Maternal and Newborn Health Care Practices in 101 Rural Districts: A Dose-Response Study. PLoS One. 2013; 8(6). DOI: 10.1371/journal.pone.0065160 PMC3672192 23750240

[18] Debelew GT, Afework MF and Yalew AW. Factors affecting birth preparedness and complication readiness in Jimma Zone, Southwest Ethiopia: A multilevel analysis. Pan Afr Med J. 2014; 19: 1–14. DOI: 10.11604/pamj.2014.19.272.4244 25870727 PMC4391899

[19] Dimtsu B and Bugssa G. Assessment of knowledge and practice towards birth preparedness and complication readiness among women in Mekelle, Northern Ethiopia: Descriptive cross-sectional. Int J Pharm Sci Res. 2014; 5(10): 4293–4301. DOI: 10.13040/IJPSR.0975-8232.5(10).4292-01

[20] Kaso M and Addisse M. Birth preparedness and complication readiness in Robe Woreda, Arsi Zone, Oromia Region, Central Ethiopia: A cross-sectional study. Reprod Health. 2014; 11(1): 55 DOI: 10.1186/1742-4755-11-55 25038820 PMC4118259

[21] Markos D and Bogale D. Birth preparedness and complication readiness among women of child bearing age group in Goba woreda, Oromia region, Ethiopia. BMC Pregnancy Childbirth. 2014; 14(1): 282 DOI: 10.1186/1471-2393-14-282 25132227 PMC4148918

[22] Bitew Y, Awoke W and Chekol S. Birth preparedness and complication readiness practice and associated factors among pregnant women, Northwest Ethiopia. Int Sch Res Not. 2016; 2016: 1–8. DOI: 10.1155/2016/8727365 PMC5046014 27722201

[23] Gebre M, Gebremariam A and Abebe TA. Birth preparedness and complication readiness among pregnant women in Duguna Fango District, Wolayta Zone, Ethiopia. PLoS One. 2015; 10(9): e0137570 DOI: 10.1371/journal.pone.0137570 26379231 PMC4574761

[24] Andarge E, Nigussie A and Wondafrash M. Factors associated with birth preparedness and complication readiness in Southern Ethiopia: A community based cross-sectional study. BMC Pregnancy Childbirth. 2017; 17(1): 412 DOI: 10.1186/s12884-017-1582-3 29216830 PMC5721538

[25] Feyissa TR and Genemo GA. Determinants of institutional delivery among childbearing age women in Western Ethiopia, 2013: Unmatched case control study. PLoS One. 2014; 9(5): 1–7. DOI: 10.1371/journal.pone.0097194 PMC4014613 24810609

[26] Tura G, Afework M and Yalew A. The effect of birth preparedness and complication readiness on skilled care use: A prospective follow-up study in Southwest Ethiopia. Reprod Health. 2014; 11(1): 60 DOI: 10.1186/1742-4755-11-60 25091203 PMC4127036

[27] Belda SS and Gebremariam MB. Birth preparedness, complication readiness and other determinants of place of delivery among mothers in Bale Zone, Goba District, South East Ethiopia. BMC Pregnancy Childbirth. 2016: 1–12. DOI: 10.1186/s12884-016-0837-8 27053241 PMC4822318

[28] Lakew Y, Tessema F and Hailu C. Birth Preparedness and Its Association with Skilled Birth Attendance and Postpartum Checkups among Mothers in Gibe Wereda, Hadiya Zone, South Ethiopia. J Environ Public Health. 2016; 2016(1994): 12–14. DOI: 10.1155/2016/6458283 PMC5222997 28115949

[29] Asseffa NA, Bukola F and Ayodele A. Determinants of use of health facility for childbirth in rural Hadiya zone, Southern Ethiopia. BMC Pregnancy Childbirth. 2016; 16(1): 1–9. DOI: 10.1186/s12884-016-1151-1 27852239 PMC5112737

[30] Wilunda C, Quaglio G, Putoto G, et al. Determinants of utilisation of antenatal care and skilled birth attendant at delivery in South West Shoa Zone, Ethiopia: A cross-sectional study. Reprod Health. 2015; 12(1): 1–12. DOI: 10.1186/s12978-015-0067-y 26432298 PMC4592558

[31] Stürmer T, Wyss R, Glynn RJG and Brookhart MA. Experimental study designs. J Intern Med. 2014; 275(6): 570–580. DOI: 10.1111/joim.12197 24520806 PMC4037382

[32] Bintabara D, Mohamed MA, Mghamba J, Wasswa P and Mpembeni RN. Birth preparedness and complication readiness among recently delivered women in Chamwino district, central Tanzania: A cross sectional study. Reprod Health. 2015; 12(1): 1–8. DOI: 10.1186/s12978-015-0041-8 25981513 PMC4447013

[33] Ministry of Health [Ethiopia]. Basic Emergency Obstretic & Newborn Care (BEmONC) Training Manual. 2006: e9–e15.

[34] Nawal D and Goli S. Birth Preparedness and Its Effect on Place of Delivery and Post-Natal Check-Ups in Nepal. PLoS One. 2013; 8(5). DOI: 10.1371/journal.pone.0060957 PMC3655026 23690921

[35] Koblinsky M, Mclaurin K and Russell-Brown P. Indicators for Reproductive Health Program Evaluation Final Report of the Subcommittee on Safe Pregnancy; 1995 (12).

[36] Stuart EA. Matching methods for causal inference: A review and a look forward. 2010; 25(1): 1–21. DOI: 10.1214/09-STS313 PMC2943670 20871802

[37] Dugoff EH, Schuler M and Stuart EA. Generalizing observational study results: Applying propensity score methods to complex surveys. Health Serv Res. 2014; 49(1): 284–303. DOI: 10.1111/1475-6773.12090 23855598 PMC3894255

[38] StataCorp LP. Stata Survey Data Reference Manual 13 edition. College Station, TX; 2013 http://www.uio.no/tjenester/it/forskning/statistikk/hjelp/brukerdokumentasjon/stata-11_svy.pdf.

[39] Assefa Y, Alebachew A, Lera M, Lynen L, Wouters E and Van Damme W. Scaling up antiretroviral treatment and improving patient retention in care: Lessons from Ethiopia, 2005–2013. Global Health. 2014; 10(1): 43 DOI: 10.1186/1744-8603-10-43 24886686 PMC4046386

[40] Magoma M, Requejo J, Campbell O, Cousens S, Merialdi M and Filippi V. The effectiveness of birth plans in increasing use of skilled care at delivery and postnatal care in rural Tanzania: A cluster randomised trial. Trop Med Int Health. 2013; 18(4): 435–443. DOI: 10.1111/tmi.12069 23383733

[41] Karkee R, Lee AH and Binns CW. Birth preparedness and skilled attendance at birth in nepal: Implications for achieving millennium development goal 5. Midwifery. 2013; 29(10): 1206–1210. DOI: 10.1016/j.midw.2013.05.002 23751594

[42] Agarwal S, Sethi V, Srivastava K, Jha PK and Baqui AH. Birth preparedness and complication readiness among slum women in Indore city, India. J Heal Popul Nutr. 2010; 28(4): 383–391. DOI: 10.3329/jhpn.v28i4.6045 PMC2965330 20824982

[43] Moran AC, Sangli G, Dineen R, Rawlins B, Yaméogo M and Baya B. Birth-preparedness for maternal health: Findings from Koupéla district, Burkina Faso. J Heal Popul Nutr. 2006; 24(4): 489–497. PMC3001153 17591346

[44] Kabakyenga JK, Östergren PO, Turyakira E and Pettersson KO. Influence of birth preparedness, decision-making on location of birth and assistance by skilled birth attendants among women in South-Western Uganda. PLoS One. 2012; 7(4). DOI: 10.1371/journal.pone.0035747 PMC3338788 22558214

[45] Brazier E, Fiorentino R, Barry S, Kasse Y and Millimono S. Rethinking How to Promote Maternity Care-Seeking: Factors Associated With Institutional Delivery in Guinea. Health Care Women Int. 2014; 35(7–9): 878–895. DOI: 10.1080/07399332.2014.916293 24821280 PMC4160277

[46] Shiferaw S, Spigt M, Godefrooij M, Melkamu Y and Tekie M. Why do women prefer home births in Ethiopia? BMC Pregnancy Childbirth. 2013; 13: 5 DOI: 10.1186/1471-2393-13-5 23324550 PMC3562506

[47] Callaghan-Koru JA, Seifu A, Tholandi M, de Graft-Johnson J, Daniel E, Rawlins B, … and Baqui AH. Newborn care practices at home and in health facilities in 4 regions of Ethiopia. BMC pediatrics. 2013; 13(1): 198 DOI: 10.1186/1471-2431-13-198 24289501 PMC4219496

[48] Callaghan-Koru JA, Estifanos AS, Sheferaw ED, de Graft-Johnson J, Rosado C, Patton-Molitors R, … and Baqui A. Practice of skin-to-skin contact, exclusive breastfeeding and other newborn care interventions in Ethiopia following promotion by facility and community health workers: Results from a prospective outcome evaluation. Acta Paediatrica. 2016; 105(12): e568–e576. DOI: 10.1111/apa.13597 27644765

