# Supplementary material for: Effect of Birth Preparedness on Institutional Delivery in Semiurban Ethiopia: A Cross-Sectional Study
Source: Ann Glob Health. 2019 Mar 21;85(1):44. doi: 10.5334/aogh.920 (PMC6634364; doi:10.5334/aogh.920)
Supplement: Supplemental Table 1. — Definitions of Birth Preparedness in Previous Studies. [file agh-85-1-920-s1.pdf]

Supplemental Table 1. Definitions of Birth Preparedness in Previous Studies

Citation	Items Included in Index/Scale	Definition of Birth Preparedness
Hiluf M, Fantahun M. Birth Preparedness and Complication Readiness among women in Adigrat town, north Ethiopia. Ethiop J Heal Dev. 2008;22(1):14-20.	• Identified a place of delivery<br>• Saved money<br>• Identified a means of transport to a place of childbirth or for the time of obstetric emergencies ahead of childbirth	• Reported all three items (100%)
Agarwal S, Sethi V, Srivastava K, Jha PK, Baqui AH. Birth preparedness and complication readiness among slum women in Indore city, India. *Journal of health, population, and nutrition*. 2010;28(4):383-391.	• Identified trained birth attendant<br>• Saved money<br>• Identified transport<br>• Identified health facility	• Reported ≥3 out of 4 items (75%)
Hailu M, Gebremariam A, Alemseged F, Deribe K. Birth preparedness and complication readiness among pregnant women in Southern Ethiopia. PLoS One. 2011;6(6). doi:10.1371/journal.pone.0021432.	• Arranged for transportation<br>• Saved money for delivery<br>• Identified skilled attendant to assist at birth<br>• Identified a health facility for emergency<br>• Identified blood donor in case of emergency.	• Reported ≥2 out of 5 items (40%)
Kabakyenga JK, Östergren PO, Turyakira E, Pettersson KO. Influence of birth preparedness, decision-making on location of birth and assistance by skilled birth attendants among women in South-Western Uganda. PLoS One. 2012;7(4). doi:10.1371/journal.pone.0035747.	• Identified skilled health professional<br>• Saved money<br>• Identified transport<br>• Had delivery kit/materials.	• Reported ≥3 of 4 items (75%)
Nawal D, Goli S. Birth Preparedness and Its Effect on Place of Delivery and Post-Natal Check-Ups in Nepal. PLoS One. 2013;8(5). doi:10.1371/journal.pone.0060957.	• Saved money<br>• Arranged for transport<br>• Identified blood donor<br>• Identified health worker<br>• Prepared delivery kit<br>• Prepared food<br>• Prepared clothes<br>• Made other preparations	• Used PCA to create scale with four categories: no preparations, poor, moderate, well prepared
Debelew GT, Afework MF, Yalew AW. Factors affecting birth preparedness and complication readiness in Jimma Zone, Southwest Ethiopia: a	• Plan where to give birth in a health facility<br>• Plan for skilled birth attendant<br>• Plan to save money	• Reported ≥3 of 5 items (60%)

multilevel analysis. Pan Afr Med J. 2014;19:1-14. doi:10.11604/pamj.2014.19.272.4244.	• Plan for transportation<br>• Identification of compatible blood donors in case of emergency	
Markos D, Bogale D. Birth preparedness and complication readiness among women of child bearing age group in Goba woreda, Oromia region, Ethiopia. BMC Pregnancy Childbirth. 2014;14(1):282. doi:10.1186/1471-2393-14-282.	• Identified place of delivery<br>• Planned for a skilled assistant during delivery<br>• Saved money for obstetric emergency<br>• Planned of mode of transport to place of delivery during emergency<br>• Planned for a blood donor during obstetric emergency<br>• Detected early signs of emergency<br>• Identifying institution with 24 hour emergency obstetric care services	• Reported ≥4 of 7 items (57%)
Kaso M, Addisse M. Birth preparedness and complication readiness in Robe Woreda, Arsi Zone, Oromia Region, Central Ethiopia: a cross-sectional study. Reprod Health. 2014;11(1):55. doi:10.1186/1742-4755-11-55.	• Identified place of delivery<br>• Saved money<br>• Identify skilled provider at birth<br>• Identified a means of transport to place of childbirth or for the time of obstetric emergencies ahead of childbirth	• Reported ≥3 of 4 items (75%)
Tura G, Afework M, Yalew A. The effect of birth preparedness and complication readiness on skilled care use: a prospective follow-up study in Southwest Ethiopia. Reprod Health. 2014;11(1):60. doi:10.1186/1742-4755-11-60.	• Planned to save money<br>• Planned to arrange transport<br>• Planned to give birth in health facility<br>• Planned to be attended by skilled attendant<br>• Planned to arrange blood donor	• Reported ≥3 of 5 criteria (60%)
Bintabara D, Mohamed MA, Mghamba J, Wasswa P, Mpembeni RNM. Birth preparedness and complication readiness among recently delivered women in chamwino district, central Tanzania: a cross sectional study. *Reproductive Health*. 2015;12(1):44.	• Knew the expected date of delivery (EDD)<br>• Identified a skilled birth attendant or health facility for delivery/emergency<br>• Identified mode of transport for delivery and obstetric emergency<br>• Saved money<br>• Identified two compatible blood donors	• Reported ≥3 of 5 items (60%)
Wilunda C, Quaglio G, Putoto G, et al. Determinants of utilisation of antenatal care and	• Identified transport,<br>• Saved money	• Reported ≥2 of 5 items (40%)

skilled birth attendant at delivery in South West Shoa Zone, Ethiopia: A cross sectional study. Reprod Health. 2015;12(1):1-12. doi:10.1186/s12978-015-0067-y.	• Identified a blood donor<br>• Decided on the facility where the baby will be born<br>• Identified a skilled birth attendant	
Gebre M, Gebremariam A, Abebe TA. Birth Preparedness and Complication Readiness among Pregnant Women in Duguna Fango District, Wolayta Zone, Ethiopia. PLoS One. 2015;10(9):e0137570. doi:10.1371/journal.pone.0137570.	• Identified a trained birth attendant<br>• Identified ahealth facility for emergency;<br>• Identified mode of transport for<br>• Delivery and/or for obstetric emergency<br>• Saved money<br>• Identified blood donor	• Reported ≥3 of 5 items (60%)
Belda SS, Gebremariam MB. Birth preparedness , complication readiness and other determinants of place of delivery among mothers in Bale Zone , Goba District , South East Ethiopia. BMC Pregnancy Childbirth. 2016:1-12. doi:10.1186/s12884-016-0837-8.	• Identified place of delivery<br>• Identified skilled birth attendant<br>• Saved money<br>• Identified means of emergency<br>• transport<br>• Arranged a blood donor for emergency<br>• Identified emergency signs<br>• Identified health institution with 24 h EmOC	• Not defined
Bitew Y, Awoke W, Chekol S. Birth Preparedness and Complication Readiness Practice and Associated Factors among Pregnant Women, Northwest Ethiopia. Int Sch Res Not. 2016;2016:1-8. doi:10.1155/2016/8727365.	• Identify place of delivery<br>• Saved money<br>• Identified skilled provider<br>• Aware of key danger signs and act on immediately,<br>• Designated decision maker<br>• Identified source of support in case of emergency<br>• “Arranged transport, EMOC, blood donor, and emergency”	• Reported ≥3 of 7 items (43%)
Lakew Y, Tessema F, Hailu C. Birth Preparedness and Its Association with Skilled Birth Attendance and Postpartum Checkups among Mothers in Gibe Wereda, Hadiya Zone, South Ethiopia. J Environ Public Health. 2016;2016(1994):12-14. doi:10.1155/2016/6458283.	• Identified skilled attendant<br>• Identified health institution for delivery and complication<br>• Saved money<br>• Arranged transportation<br>• Identified blood donor<br>• Arranged essentials (clean clothes,	• Used principal components analysis (PCA) to develop a scale with three categories: poor, moderate, and well prepared.

	delivery kit)<br>• Arranged food<br>• Made other arrangements	
Zepre K, Kaba M. Birth preparedness and complication readiness among rural women of reproductive age in Aabeshige district, Gguraghe zone, SNNPR, Ethiopia. Int J Womens Health. 2017;9:11-21. doi:10.2147/IJWH.S111769.	• Identified place of delivery<br>• Identified skilled health care provider<br>• Saved money<br>• Identified transport ahead of emergency<br>• Identified a blood donor	• Reported ≥3 of 5 items (60%)
Andarge E, Nigussie A, Wondafrash M. Factors associated with birth preparedness and complication readiness in Southern Ethiopia: a community based cross-sectional study. BMC Pregnancy Childbirth. 2017;17(1):412. doi:10.1186/s12884-017-1582-3	• Saved money (planned to)<br>• Identified health facility (planned to) for delivery (health center/hospital)<br>• Identified skilled provider at birth (planned to)<br>• Identified a means of transport to place of childbirth or for the time of obstetric emergencies (planned to) ahead of childbirth	• Reported ≥3 of 4 items (75%)
